# Hepatosplenic Gamma Delta T-Cell Lymphoma (HSGDTCL): Two Rare Case Reports from Western India 

**Published:** 2017-10-01

**Authors:** Irappa Madabhavi, Gaurang Modi, Harsha Panchal, Apurva Patel, Swaroop Revannasiddaiah, Asha Anand, Sonia Parikh, Kshitij Joshi, Malay Sarkar

**Affiliations:** 1 Department of Medical and Pediatric Oncology, GCRI, Ahmedabad, Gujarat, India; 2 Department of Radiotherapy, Government Medical College, Haldwani, Uttarakhand, India; 3 Department of Pulmonary Medicine, IGMC, Shimla, Himachal Pradesh, India

**Keywords:** Hepatosplenic gamma delta T cell lymphoma (HSGDTCL), Immuno competent patients, CHOP

## Abstract

Peripheral T cell lymphomas are a heterogeneous group of post-thymic, mature lymphoid malignancies, accounting for approximately 10-15% of all non-Hodgkin's lymphomas. Hepatosplenic T-cell lymphoma (HSGDTCL) is a rare entity, which is characterized by primary extra nodal disease with typical sinusoidal or sinusal infiltration of the liver and the spleen, respectively by expression of the T-cell receptor γδ chain, and by a number of other frequent clinicopathologic features, including aggressive course of disease. Secondary involvement of liver by hematopoietic malignancies is much more common as compared to primary liver involvement. Primary involvement of liver by non- Hodgkin’s lymphoma (NHL) is documented and mostly DLBCL (diffuse large B cell lymphoma) type. But, T cell lymphoma primarily arising from liver is very rare. It occurred commonly in immunocompromised patients and prognosis is very poor. Here, we present two case reports of Hepatosplenic gamma-delta T-cell lymphoma (HSGDTCL) and both are immunocompetent patients. Liver biopsy from the mass and subsequent IHC (immunohistochemistry) were performed for the purpose of diagnosis, which were positive for LCA (leukocyte common antigen), CD2 and negative for CD5, CD20 and CD79a. First patient was a 63-year-old female with hepatitis C virus seropositivity presented with liver mass simulating hepatocellular carcinoma. Second patient was a 60-year- old male, chronic alcoholic patient, presented with liver mass and lytic bony lesion in pelvis. Both patients were managed with conventional CHOP (cyclophosphamide, hydroxydaunorubicin, vincristine, and prednisolone) and showed complete response after 4 cycles of chemotherapy. After completion of 6 cycles of chemotherapy, both patients remained under 6-month surveillance period for any recurrence of the disease.

## Introduction

 Hepatosplenic T-cell lymphoma (HSGDTCL) is a rare and aggressive extra nodal lymphoma derived mostly from cytotoxic γδ T-cells. The peak incidence is in adolescents and young adults, and is more common in males. Up to 20% of HSGDTCL arise in the setting of chronic immune suppression, most commonly solid organ transplantation or prolonged antigenic stimulation.

Typical features are sinusoidal infiltration of the liver and sinusal infiltration of the spleen, no lympadenopathy, an aggressive clinical course, and a predominance of young male adults. Despite use of a variety of treatment regimens including bone marrow or peripheral stem cell transplantation, it has not been possible to establish an effective treatment. HSGDTCL subtype is exceedingly rare and only few cases are reported in the world literature^1^. High index of suspicion and expert pathologist reporting requires to diagnose this rare entity. The term HSGDTCL was initially included in the Revised European-American Lymphoma (REAL) classification^2^. As it has distinct histology, clinical manifestations and immunophenotypic (IHC) features, it is included as Hepatosplenic T-cell lymphoma in the current World Health Organization classification.

## Case reports

**Case 1**

**Figure1:**
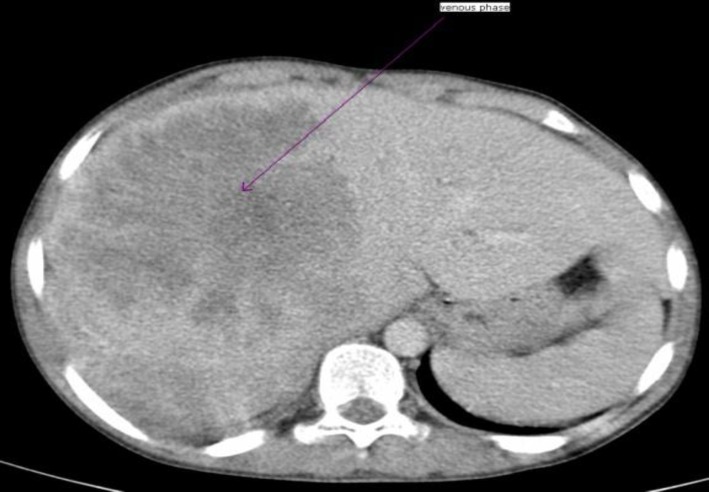
Ill- defined soft tissue density lesion of size 12x10 cm in right lobe of liver, which shows marked heterogeneous enhancement.

A 63-year-old female presented to our cancer center following a 2-month history of progressive abdominal distension, anorexia and weakness. On clinical examination, moderate non-tender hepatosplenomegaly, moderate ascites, and icterus tinge in sclera were found. No palpable lymphadenopathy was found clinically. Hemogram showed moderate anemia with thrombocytopenia. There was no evidence of atypical lymphoid cells in peripheral blood. She was recently diagnosed with Hepatitis C infection, but did not receive treatment. Computed Tomography of the abdomen showed an ill-defined soft tissue density lesion (12x10 cm) in right lobe of liver, which showed marked heterogeneous enhancement (Figure1).

**Figure 2:**
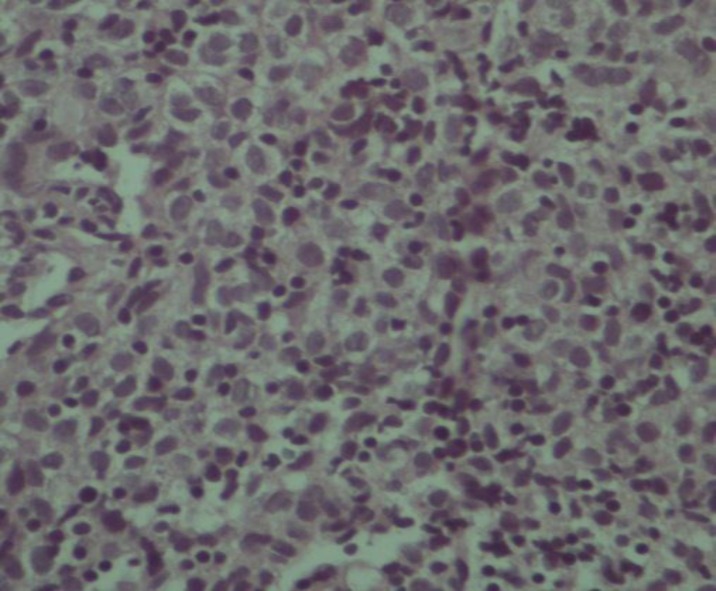
Shows lymphocytic infiltration of cells in variably dilated sinusoids. Neoplastic cells are small in size with hyper chromatic nuclei and prominent nucleoli as well as scanty cytoplasm

Serum Alfa Feto Protein (AFP) marker was 16 IU/ml. USG guided biopsy from liver lesion was done and showed lymphocytic infiltration of cells in variably dilated sinusoids. Neoplastic cells are small in size with hyper chromatic nuclei, conspicuous nucleoli and scanty cytoplasm (Figure 2). 

**Figure 3:**
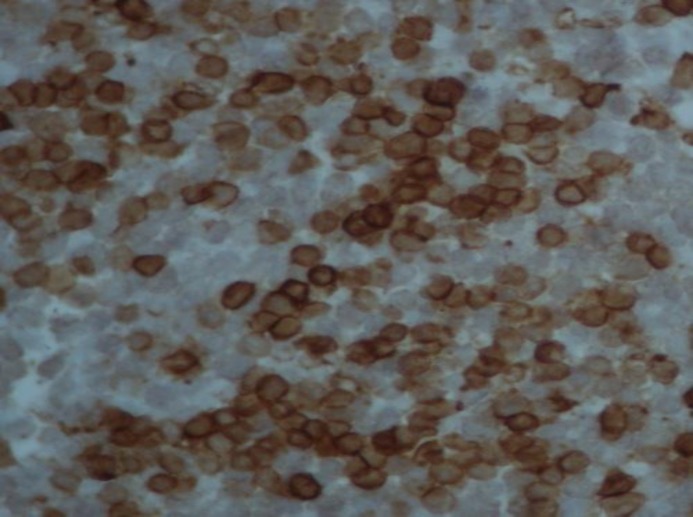
LCA positivity

**Figure 4:**
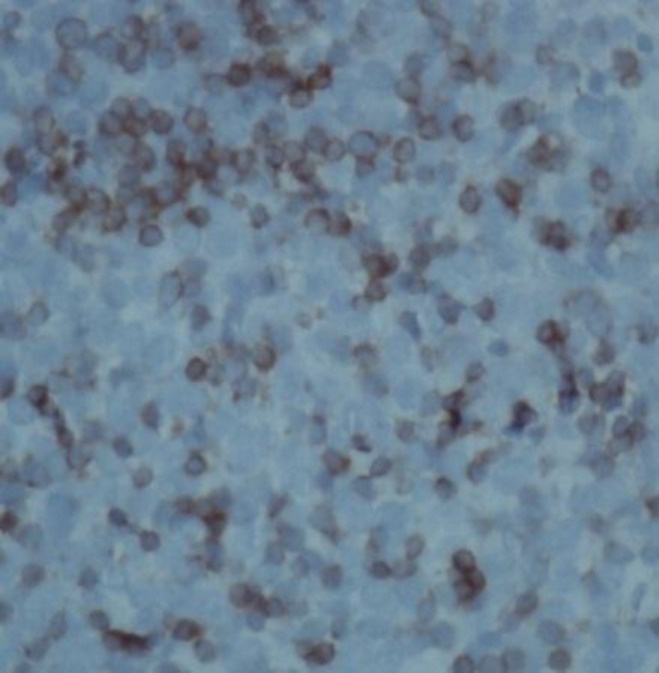
CD2 positivity

Immunohistochemistry (IHC) showed positivity for Leukocytes Common Antigen (LCA) (Figure. 3) and T cell markers like CD2 and negativity to CD 20 (pan B marker) (Figure 4), CD 30 and CD79a, suggesting primary hepatosplenic gamma delta T-cell lymphoma (HSGDTCL). For staging workup, whole body CT scan was done and no abnormality except liver lesion was detected. Marrow aspiration and biopsy showed normocellular marrow uninvolved by malignancy. She was offered mini CHOP due to her poor performance status and age. She was given cyclophosphamide with flat dose of 500mg, doxorubicin with flat dose of 50mg, vincristine 2mg/m^2^, oral prednisolone 60mg/m^2^ (for 5 days). She tolerated chemotherapy very well, and after 4 cycles imaging showed complete response, and then she completed a total 6 cycles of chemotherapy. She is under regular surveillance at our clinic for any recurrence of the disease. 

**Case2**

A 60-year-old male chronic alcoholic patient presented with a 2-month history of severe weight loss followed by jaundice and abdominal pain. On clinical examination, no palpable lymphadenopathy and hepatosplenomegaly were noted. Meanwhile, his performance score was 1. CT scan showed 6x7 cm mass lesion in left lobe of liver with lytic bone lesion in pelvis bone. Laboratory investigations revealed anaemia, thrombocytopenia, increased bilirubin (obstructive type), and altered coagulation profile (prolong prothrombin and activated partial thromboplastin time). We gave him symptomatic care with vitamin K, cryoprecipitate and fresh frozen plasma. After normalization of coagulation profile, ultrasonography-guided liver biopsy was performed without any acute complications. Histopathology and Immunohistochemistry findings were consistent with the diagnosis of HSGDTCL. He was managed with conventional CHOP (cyclophosphamide hydroxydaunorubicin, vincristine, and prednisolone). The patient showed complete response after 4 cycles of chemotherapy and completed a total 6 cycles of chemotherapy. We now have had this patient under surveillance for 6 months to detect disease recurrence. 

## Discussion

 Hepatosplenic gamma delta T-cell lymphoma (HSGDTCL) is a rare subtype of lymphoid neoplasm from the heterogeneous PTCLs (peripheral T cell lymphoma) group. Tumor cells show expression of gamma/delta-TCR and presence of gamma/delta-TCR gene rearrangements. Being the rare entity true incidence of HSGDTCL is still unknown. Median age of incidence is 32 years; males are most commonly affected gender. It probably distributed worldwide without any racial predisposition^1^ and both patients in our reports were over 60 years. 

Usually, HSGDTCL occurs in adult males, often accompanied by systemic symptoms, a marked hepatosplenomegaly in the absence of lymphadenopathy. While splenomegaly is a constant finding, hepatomegaly could lack in 10–15% of cases. Abnormalities in liver function tests are present in half of cases, with mildly elevated AST, ALT and alkaline phosphatise levels. Lactate dehydrogenase level is usually markedly elevated (up to 3000 U/L). Anemia is present in 75% of cases with a hematocrit ranging from 20% to 28%. Thrombocytopenia is observed in 85% and seems correlated to the clinical evolution of the disease. Bone marrow involvement is almost constant at diagnosis, and circulating neoplastic lymphocytes are present in peripheral blood of 50–80% of cases. Patients present with systemic symptoms (fever), abdominal pain, weakness, and marked hepatosplenomegaly in the absence of lympadenopathy. Other findings are easy bruising and purpura^3^.

The most common histopathological feature present in almost all patients with hepatosplenic Ύδ T cell lymphoma is infiltration by malignant cells of the spleen and frequently of the liver. Bone marrow involvement is seen in approximately two-thirds of the patients at diagnosis, more often in progressive course of disease, lympadenopathy is rare and sometimes associated with infiltration of other organs by malignant cells in the terminal stage of disease. HSGDTCL is characterized by a monotonous neoplastic infiltrate consisting of medium-sized lymphocytes with a moderate amount of cytoplasm and indistinct cellular borders. Malignant Hepatosplenic Ύδ T cells are usually of small to medium size with relatively regular or folded nuclei, mostly inconspicuous nucleoli, and with a mature dispersed chromatin and a pale blue, not granulated somewhat abundant cytoplasm. These cells usually involve the spleen, liver, bone-marrow, and peripheral blood. Blast-like appearance at diagnosis is rare, however, terminal transformation to blast-like cells with large nucleoli has been reported several times.^4^

In the past, HSGDTCL diagnosis was usually made on splenectomy. Presently, an accurate diagnosis can be made on bone marrow histology with immunophenotypic evaluation, liver biopsy and/or cytological and immunocytochemical examinations of peripheral blood, avoiding unnecessary and possibly harmful surgical procedures.^5^ Diagnosis of HSGDTCL is challenging as IHC is required in confirming the disease. A phenotype which has been accepted to be common in Hepatosplenic Ύδ T cell lymphoma is CD2^+^, CD3^+^, CD4^-^, CD5^-^, CD7^+^, CD8^- ^and^ TCR-^ Ύδ^ +^. TCR-Vδexpression seems to be restricted to Vδ1. NK-related antigens CD16 and CD56 are frequently expressed. Other molecules associated with activation but not strictly restricted to T cells such as CD11b, CD11c, CD38, CD43, and Fas ligand are frequently expressed. B cell markers (CD19, CD20, CD21, and CD22), immunoglobulin’s, TCR-ᶏβ chain, TdT, CD10, CD15, CD25, CD33, CD34, CD41, and CD68 were consistently negative. As may be expected, there are many exceptions to this 'common phenotype' since expression of CD5, CD7, CD8, CD16 and CD56 is variable. Also, loss of CD3 expression associated with disease prognosis.^6^ Main differential diagnosis is NK cell lymphoma and NK-like T-cell lymphomas. CD11b, CD16, and CD56, markers for NK cells, may be falsely positive in HSGDTCL. In that case, CD57 would probably help to diagnose the NK cell lymphoma.^7^

Earlier studies showed the presence of an isochromosome 7q, often associated with trisomy 8, inspiring the discussion that isochromosome 7q may represent the primary chromosomal aberration accompanied by trisomy 8 as a secondary change. Meanwhile, isochromosome 7q was detected in 13 patients, all of whom were male, and associated with trisomy 8 in 10 cases; however, trisomy 8 without isochromosome 7 was not reported. Although isochromosome 7q and trisomy 8 by themselves are known cytogenetic abnormalities in a number of hematological malignancies and solid tumours, the combination of both seems to be unique in Hepatosplenic Ύδ T cell lymphoma^8^.

Differential diagnosis includes hepatotropic virus, acute liver infection and myelodysplastic syndrome in patients who present non-involved bone marrow hyperplasia; autoimmune thrombocytopenic purpura in cases with isolated thrombocytopenia; acute lymphoblastic leukaemia in patients with a high number of circulating blast cells; and T-cell lymphoblastic lymphoma expressing Ύδ TCRs. 

Chronic antigenic stimulation is associated with the expansion of T cells. It occurrs in various conditions like patient on long-term immunosuppressive therapy, falciparum malaria, and autoimmune disorders such as rheumatoid arthritis, celiac sprue, polymyositis, multiple sclerosis, and systemic lupus erythematosus. Isochromosome arm 7q is the hallmark of this type of lymphoma, probably due to genetic changes derived by reactive T cells and detected by FISH, not by conventional cytogenetics^9^.

Pancytopenia was the initial presentation for most of the patients and splenomegaly was the cause presumed. Incidence of thrombocytopenia was much more common than other cell lines. Cytokine secretion by neoplastic GD T Cell cells such as interferon-gamma might also contribute to the suppression of hematopoiesis^10^^,^^11^. Previously splenectomy was done to cure pancytopenia. Weidmann demonstrated that bone marrow involvement in HSGDTCL is found in approximately two-thirds of patients at diagnosis^12^. In our two cases, we retrospectively examined the marrow and bone biopsy material but could found any involvement by lymphoma. The standard staging system used for HSGDTCL is the same as that proposed for Hodgkin’s disease at the Ann Arbor Conference in 1971. According to this staging system, HSGDTCL constitutes by definition a stage III–IV disease, often with systemic symptoms (stage B; fever of unknown origin, night sweats and weight loss of more than 10% of body weight), while the presence of a bulky mass, such as a lesion of 10 cm or more in the longest diameter, is rare.

HSGDTCL exhibits a marked chemo resistance to currently used regimens, a rapidly progressive behaviour, and dismal prognosis.  Patients with post-transplant HSGDTCL exhibit an especially poor outcome. A standard option for patients with HSGDTCL has not been yet established because of the extreme rarity of this clinical entity, the difficulty of assessing the origin and disparate reports in the literature including small case series treated with various therapies. CHOP (cyclophosphamide, doxorubicin, vincristine, prednisone), Pentostatin, Liposomal ATRA (all trans retinoic acid), DHAP (dexamethasone, cytarabine, cisplatin), MINE (mesna, ifosfamide, mitoxantrone, etoposide), Hyper CVAD (fractionated cyclophosphamide, doxorubicin, dexamethasone, vincristine), HyperCVIDD, fractionated cyclophosphamide, liposomal doxorubicin, dexamethasone, vincristine), ESHAP (etoposide, methylprednisolone, cytarabine, cisplatin), autologus or allogeneic stem cell transplantation^2^.

No predictive prognostic markers yet available for prognosis and survival duration by literature review is between 0 to 72 months^12^. However, a close correlation between thrombocytopenia degree and relapse and survival has been reported.

## CONCLUSION

 HSGDTCL is a unique subtype of T cell lymphoma affecting only liver and spleen. It is clinically challenging to diagnosis and seeking help from IHC required. Though no standard guideline available to treat this aggressive type of tumour, combination of chemotherapy (CHOP like) can prolong duration of survival. As the disease is rare, its biology is not yet well understood. However, because of the dilemma of an aggressive clinical course and an unsatisfactory response to treatment, more insight in the biology of the malignant  T cells may hopefully contribute to develop new therapeutic concepts.
